# Enhanced Expression of Cystathionine β-Synthase and Cystathionine γ-Lyase During Acute Cholecystitis-Induced Gallbladder Inflammation

**DOI:** 10.1371/journal.pone.0082711

**Published:** 2013-12-09

**Authors:** Li Zhang, Chenwei Pan, Bin Yang, Yong Xiao, Baoping Yu

**Affiliations:** 1 Department of Gastroenterology, Renmin Hospital of Wuhan University, Wuhan, China; 2 Department of Infectious Disease, the Second Affiliated Hospital of Wenzhou Medical University, Wenzhou, China; University of Navarra School of Medicine and Center for Applied Medical Research (CIMA), Spain

## Abstract

**Background:**

Hydrogen sulfide (H_2_S) has recently been shown to play an important role in the digestive system, but the role of endogenous H_2_S produced locally in the gallbladder is unknown. The aim of this study was to investigate whether gallbladder possesses the enzymatic machinery to synthesize H_2_S, and whether H_2_S synthesis is changed in gallbladder inflammation during acute acalculous cholecystitis (AC).

**Methods:**

Adult male guinea pigs underwent either a sham operation or common bile duct ligation (CBDL). One, two, or three days after CBDL, the animals were sacrificed separately. Hematoxylin and eosin-stained slides of gallbladder samples were scored for inflammation. H_2_S production rate in gallbladder tissue from each group was determined; immunohistochemistry and western blotting were used to determine expression levels of the H_2_S-producing enzymes cystathionine β-synthase (CBS) and cystathionine γ-lyase (CSE) in gallbladder.

**Results:**

There was a progressive inflammatory response after CBDL. Immunohistochemistry analysis showed that CBS and CSE were expressed in the gallbladder epithelium, muscular layer, and blood vessels and that the expression increased progressively with increasing inflammation following CBDL. The expression of CBS protein as well as the H_2_S-production rate was significantly increased in the animals that underwent CBDL, compared to those that underwent the sham operation.

**Conclusions:**

Both CBS and CSE are expressed in gallbladder tissues. The expression of these enzymes, as well as H_2_S synthesis, was up-regulated in the context of inflammation during AC.

## Introduction

Acute acalculous cholecystitis (AC) is characterized by gallbladder inflammation without evidence of calculi or sludge [1]. Bile stasis, ischemia-reperfusion injury, and systemic inflammatory response syndrome (SIRS) have been suggested as pathogenic factors [2]. In critically ill patients, AC is believed to have a more fulminant course, and is associated with significantly higher morbidity and mortality than gallstone-associated acute cholecystitis [1]. Common bile duct ligation (CBDL) in the guinea pig produces a histologic picture that is nearly identical to that of acute human cholecystitis, with subserosal edema, hemorrhage, white blood cell infiltration, and vasodilation [3]. This simple animal model also reveals impaired gallbladder muscle contractility [2,3]. 

Hydrogen sulﬁde (H_2_S) has been proposed to be the third endogenous gaseous transmitter [4] in addition to nitric oxide (NO) and carbon monoxide (CO) in mammals. Endogenous H_2_S is generated from the substrate L-cysteine by two key enzymes: cystathionine β-synthase (CBS) and cystathionine γ-lyase (CSE) [5]. CBS and CSE are widely distributed in tissues; however, CBS is the main H_2_S-forming enzyme in the CNS whereas CSE is the main H_2_S-forming enzyme in the cardiovascular system [4,6]. Recent studies have demonstrated that H_2_S plays an important role in the digestive system, and that CBS and CSE are both expressed in the gastrointestinal (GI) tract of rats and mice and in the healthy human gut [7,8]. In addition, H_2_S can be produced at 0.2-3.4 mM in the GI tract of mice and humans by intestinal microbiota (sulfate-reducing bacteria) [8]. So, the GI tract contains the highest concentration of H_2_S in the human body [7,9]. However, the role of endogenous H_2_S produced locally in the gallbladder is not well understood.

H_2_S plays a key role in many physiological and pathological processes such as inflammation, contractile responses, visceral hyperalgesia, and cancer development [8]. H_2_S participates in the regulation of various GI functions, including motility control, inflammation, secretion and nociception [10]. The presence of H_2_S in the mucosa/submucosa of the colon stimulates primary afferent nerve, thus increasing chloride secretion [10]. It seems that H_2_S has either an inhibitory or excitatory effect on contractile activity of smooth muscle [11]. H_2_S has also been reported to exert both anti-inflammatory and pro-inflammatory effects [8]. Administration of NaHS, a H_2_S donor, prevented the inflammation-associated reduction of gastric mucosal blood flow and reduced acetyl salycilic acid (ASA)-induced leukocyte adherence in mesenteric venules [12]. Endogenous H_2_S or oral administration of NaHS, protected against gastric ischemia-reperfusion in rats by inhibiting oxygen free radical overproduction [13,14]. Experimental evidence supports a pro-inflammatory role for H_2_S in carrageenan-induced hindpaw edema [15], acute pancreatitis (AP) [16], lipopolysaccharide (LPS)-induced endotoxemia [17], as well as cecal ligation and puncture (CLP)-induced sepsis [18,19] . To date, there is a paucity of studies investigating the roles of endogenous H_2_S in gallbladder inflammation during AC. We hypothesized that gallbladder expresses H_2_S-synthetic enzymes, which would exhibit alterations at the expression or functional level after CBDL in the guinea pig gallbladder model. 

Therefore, the aim of the present study was to investigate whether gallbladder expresses CSE and CBS, and whether H_2_S synthesis is changed during AC. By using the CBDL model in guinea pigs, we provide evidence for the up-regulation of H_2_S synthetic enzymes, as well as H_2_S synthesis, under the conditions of increased gallbladder inflammation during AC.

## Materials and Methods

### Animals and experimental design

Adult male guinea pigs (450-550 g) were provided by the Center for Animal Experiment of Wuhan University and housed in laboratory conditions. All protocols were approved by the Institutional Animal Care and Use Committee of Wuhan University. Forty-eight guinea pigs were assigned randomly to either the sham or CBDL group. Acute acalculous cholecystitis (AC) was induced in animals by common bile duct ligation (CBDL), as described previously [3]. Brieﬂy, the animals were anesthetized with an intraperitoneal (i.p.) injection of pentobarbital sodium (40 mg/kg body weight; Sigma-Aldrich, St. Louis, MO). A laparotomy was performed and the distal end of the common bile duct was ligated (4–0 silk) with minimal manipulation of the bile duct and no manipulation of the gallbladder. The abdomen was then sutured. The sham operation (n=12) included all the surgical steps except for the common bile duct ligation. When the animals were alert, they were housed separately and were provided with food and water ad libitum. They were monitored until they were killed one to three days later. At the indicated time points (1, 2, or 3 days after the operation, n=12 per group), the guinea pigs were sacrificed with a lethal i.p. dose of pentobarbital sodium. Gallbladder tissue samples were harvested for subsequent assays.

### Histopathological analysis

Freshly-prepared gallbladder segments were fixed with 10% neutral formaldehyde, processed in paraffin, and 4 μm-thick slices were stained with hematoxylin and eosin. Three sections per animal, with at least five randomly selected fields per section, were used in histopathological analyses using light microscopy. An inflammation scoring system, described by Parkman et al [3], was used; the scored ranged from 0 (not present) to 17 (the most severe), on the basis of histologic changes. The degree of inflammatory cell infiltrate was graded as 0, 1, 2, or 3; hemorrhage (extravasation of RBCs), edema, surface ulceration, and fibroblast proliferation were graded as 0, 1, 2, or 3; vascular dilation and Rokitansky-Aschoff sinus formation, were scored as 1 if present and 0 if absent [3].

### Measurement of endogenous H_2_S production

Tissue H_2_S was measured essentially as described elsewhere [20]. Gallbladder tissue was homogenized brieﬂy. The assay mixture contained 10% (w/v) tissue homogenate. Cryovial test tubes (2 mL) were used as the center wells, each containing 0.5 mL 1% zinc acetate and a filter paper of 2 × 2.5 cm^2^. The reaction was performed in a tightly-sealed Erlenmeyer ﬂask and initiated by transferring the flasks from ice to a water bath at 37°C. The reaction was stopped after incubation for 90 min and the flasks were incubated at 37°C for another 60 min. The contents of the center wells were then transferred to test tubes, each containing 3.5 mL of water. Subsequently, N,N-dimethyl-p-phenylenediamine sulfate (20 mM; 0.5 mL) in 7.2 M HCl was added, immediately followed by FeCl_3_ (30 mM; 0.4 mL) in 1.2 M HCl. The absorbance of the resulting solution at 670 nm was measured 20 min later by spectrophotometry. The H_2_S concentration was calculated against a calibration curve of an NaHS standard. 

### Immunohistochemistry

Formalin-ﬁxed gallbladder samples were embedded in paraffin and cut into 4-μm thick sections, which were deparaffinized in xylene and rehydrated in graded ethanol. For antigen retrieval, protein antigenicity was enhanced in citrate buffer for 10-15min in a microwave. Sections were treated with 3% hydrogen peroxide for 15 min to inhibit endogenous peroxidase activity. After this procedure, the sections were incubated in a 10% blocking serum for 30 min to block nonspecific binding , and then incubated overnight at 4°Cwith a mouse anti-CBS monoclonal antibody (1:200; sc-133154, Santa Cruz Biotechnology) or a rabbit anti-CSE polyclonal antibody (1:200;sc-135203, Santa Cruz Biotechnology). After being washed with phosphate-buffered saline (PBS), the sections were incubated with biotinylated rabbit anti-mouse or horse anti-rabbit antibodies (Boster, China) at 37°C for 30 min. Next, the sections were stained in a 3-3ʹ-diaminobenzidine solution (DAB) until the brown reaction product could be visualized, and finally counterstained with hematoxylin and mounted with neutral gum. Simultaneously, negative controls were performed by substituting the primary antibody with a normal serum at the same dilution. The results were evaluated semi-quantitatively under a high-magniﬁcation (×400) light microscope.

### Western blot analysis

Gallbladder tissues were homogenized in ice-cold RIPA buffer (Beyotime Biotechnology Company, Jiangsu, China) containing 50 mM Tris (pH 7.4), 150 mM NaCl, 1% Tergitol-type NP-40, 0.5% sodium deoxycholate, 0.1% SDS, 1 mM EDTA and 2 μg/L leupeptin. The protein concentrations in the samples were determined using a BCA protein assay kit (Beyotime Biotechnology Company, Jiangsu, China). Equal amounts of proteins (50 μg) from homogenates were resolved by sodium dodecyl sulfate polyacrylamide gel electrophoresis (SDS-PAGE) in 10% gels, and then transferred to nitrocellulose membranes. Membranes were blocked in Tris-buffered saline (TBS) containing 0.1% Tween-20 (TBST) and 5% nonfat dry milk for 2 h at room temperature. The specific antibodies for CSE and CBS were purchased from Santa Cruz Biotechnology (Santa Cruz Biotechnology, Inc. Santa Cruz, CA). After washing with TBST, the membranes were incubated with primary antibodies for CBS or CSE (1:400) at 4°C overnight. After washing with TBST, the membranes were incubated with a horseradish peroxidase-conjugated anti-goat IgG secondary antibody (Boster, China) for 1 h at room temperature and further washed for 30 min with TBST. Immunoreactive proteins were visualized using a chemiluminescence western blotting detection system (Pierce Biotechnology, Rockford, IL). Glyceraldehyde 3-phosphate dehydrogenase (GAPDH) was used as an internal control.

### Statistical analysis

Data are reported as the mean ± standard deviation (SD). The inflammation scores in different groups were evaluated using the Kruskal-Wallis H test. Other laboratory data were analyzed by one-way ANOVA followed by the LSD post-hoc test. Values of *P* <0.05 was considered statistically significant. The SPSS 13.0 software was used for statistical analysis. CBDL-1, CBDL-2, and CBDL-3 refer to the groups of animals sacrificed 1, 2, and 3 days after CBDL, respectively.

## Results

### CBDL induces inflammation in the gallbladder

**Figure 1:**
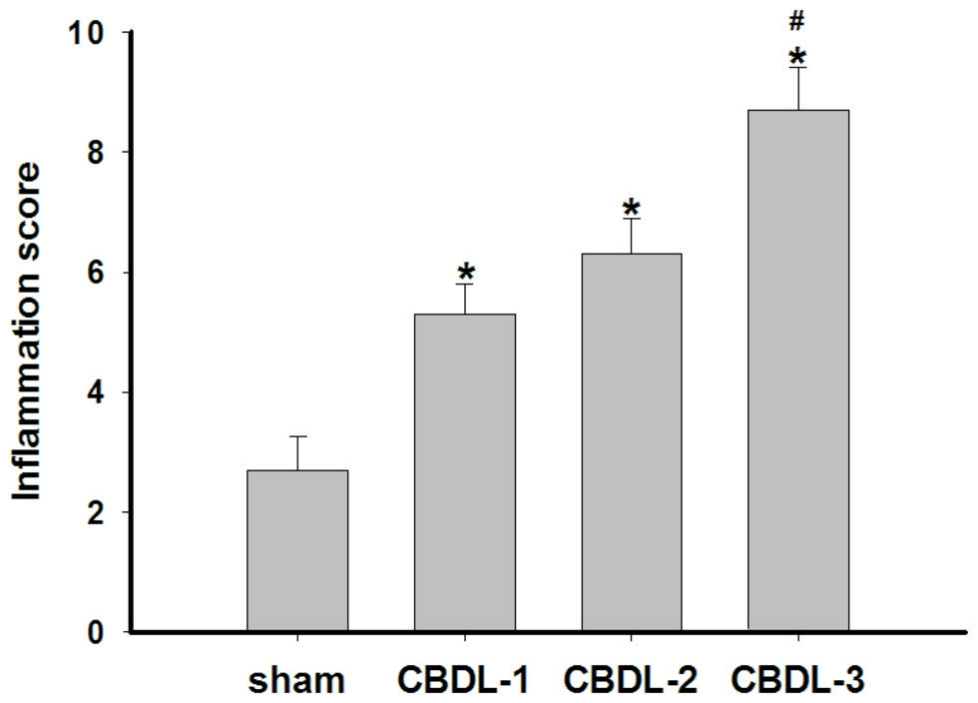
Effect of Common Bile Duct Ligation (CBDL) on Gallbladder Inflammation Scores. Data shown are as the mean ± SD (n=12/group). *P <0.05 vs. sham; #P <0.05 vs. CBDL-1 and CBDL-2.

**Figure 2:**
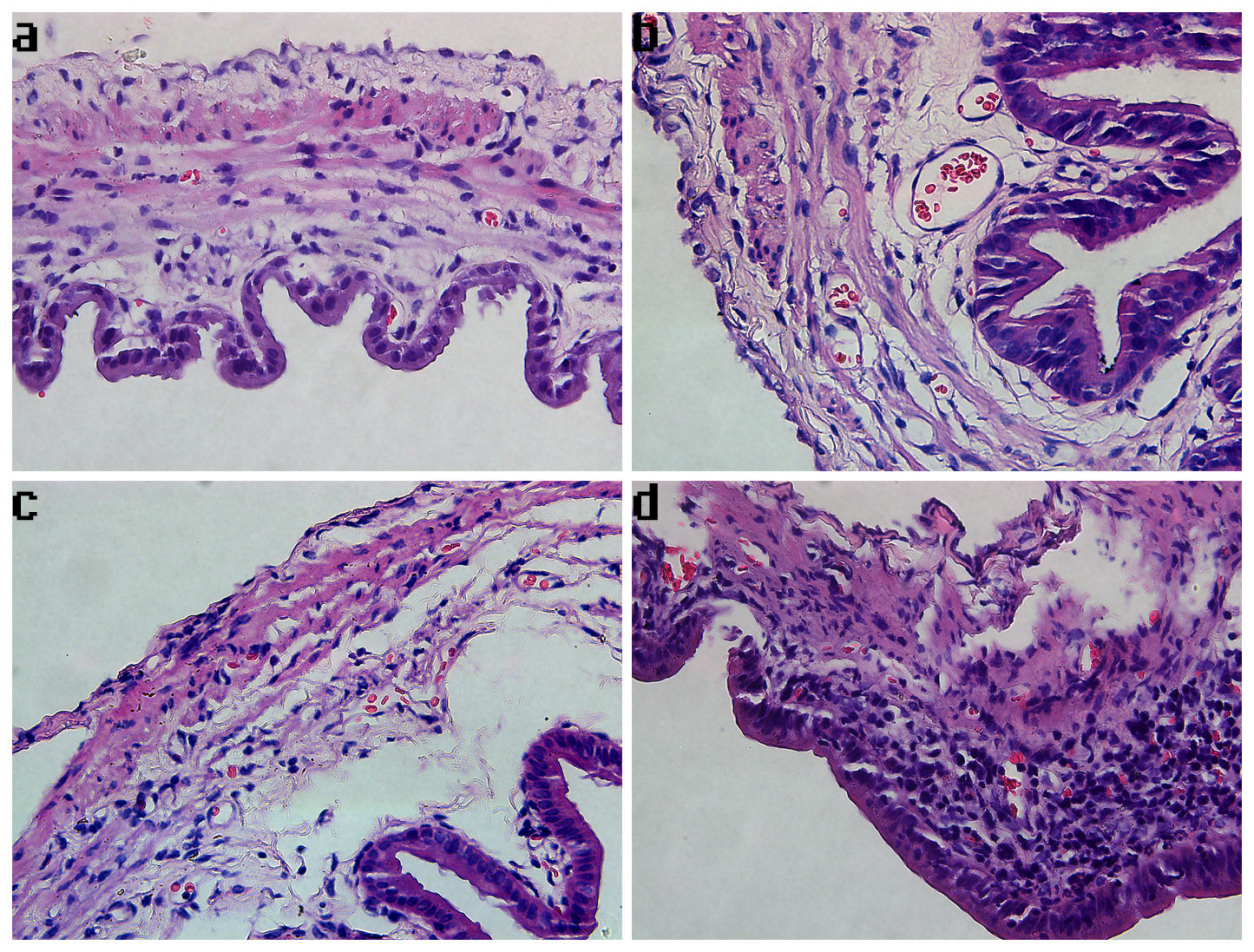
Photomicrographs of gallbladder samples stained with hematoxylin and eosin in each group. The magnification of photomicrographs is ×400. (a) Cholecystic pathology of a representative sample from the group that underwent the sham operation, showing normal gallbladder histology. (b) Cholecystic pathology of a representative sample of an animal from the CBDL-1 group, indicating edema, congestion, and a few inflammatory cells infiltrating into the mucosa. (c) Cholecystic pathology of a representative sample of an animal from the CBDL-2 group, indicating pathological changes of gallbladder resembling that of the CBDL-1 group. (d) Cholecystic pathology of a representative sample of an animal from the CBDL-3 group, exhibiting pronounced edema with significant inflammatory cell infiltrates in the lamina propria.

After CBDL, there was a progressive inflammatory response in the gallbladder (Figure 1). The most prominent inflammatory cells (primarily neutrophils) infiltrated in the lamina propria of the gallbladder wall from an animal 3 days after CBDL, with some splaying of the muscle fibers due to edema (Figure 2). The mean inflammation score in the CBDL group was higher than that of the sham surgical group (CBDL-1: 5.33 ±0.40 vs. sham: 2.67±0.57, CBDL-2: 6.21±0.32 vs. sham: 2.67±0.57, CBDL-3: 8.65±0.51 vs. sham: 2.67±0.57, n=12/group, *P* <0.05) (Figure 1). In the CBDL group, at 3 days after surgery (CBDL-3), the inflammation score was significantly elevated compared to the groups from days 1 to 2 post-ligation (CBDL-1, CBDL-2) (8.65±0.51 vs. 5.33 ±0.40 and 6.21±0.32, n=12/group, *P* <0.05). The inflammation of CBDL increased by 0.99- (or 1.30-) and 2.24-fold, respectively, after CBDL for 1 (or 2) day and CBDL for 3 days. There was no statistical difference between the inflammation scores of the sham surgical controls and the normal controls (data not shown).

### H_2_S production rate in the gallbladder

**Figure 3:**
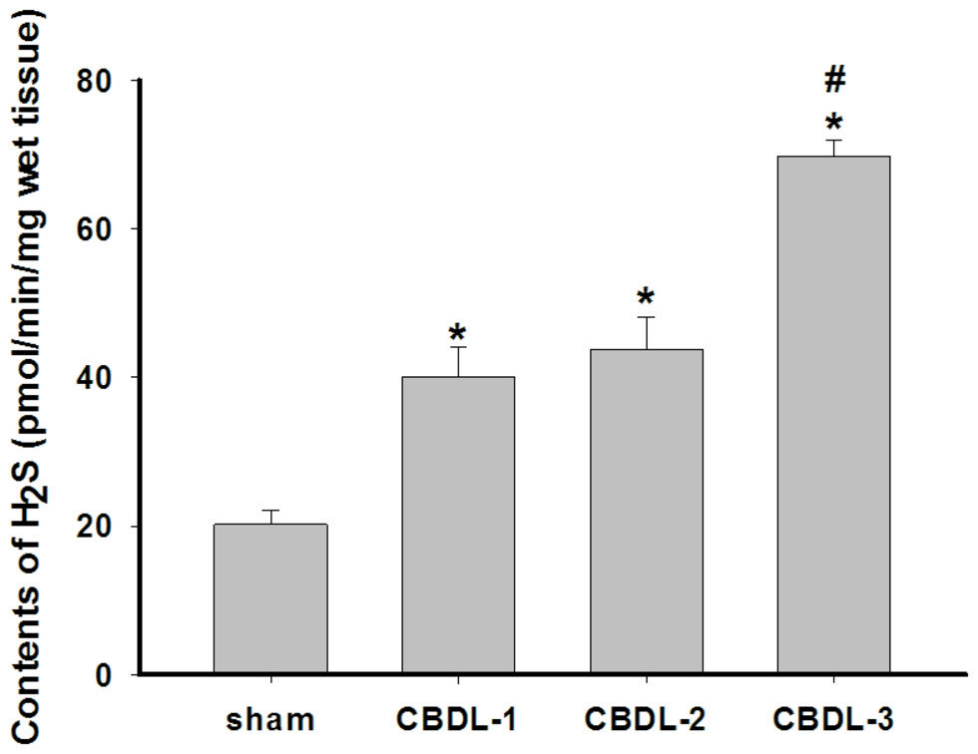
H_2_S synthesis in the gallbladder from guinea pigs. Data shown are the mean ± SD (n=12/group). * *P* <0.01 vs. sham; #P <0.01 vs. CBDL-1 and CBDL-2.

H_2_S synthesis in the gallbladder from the CBDL group was significantly increased by 0.98-(or 1.11-) and 2.45-fold in CBDL-1(or CBDL-2) and CBDL-3, respectively, compared with that of the sham group (CBDL-1: 40.13±3.94 pmol/min/mg vs. sham: 20.21±1.84 pmol/min/mg, CBDL-2: 42.79±4.39 pmol/min/mg vs. sham: 20.21±1.84 pmol/min/mg, CBDL-3: 69.83±2.09 pmol/min/mg vs. sham: 20.21±1.84 pmol/min/mg, n=12/group, *P* <0.01). There was no statistically significant difference between the CBDL-1 and CBDL-2 groups, while the endogenous level of H_2_S produced in the CBDL-3 group was markedly higher compared to that in the CBDL-1 and CBDL-2 groups (69.83±2.09 pmol/min/mg vs. 40.13±3.94 and 42.79±4.39 pmol/min/mg, n=12/group, *P* <0.01) (Figure 3).

### Localization of CBS and CSE in gallbladder

**Figure 4:**
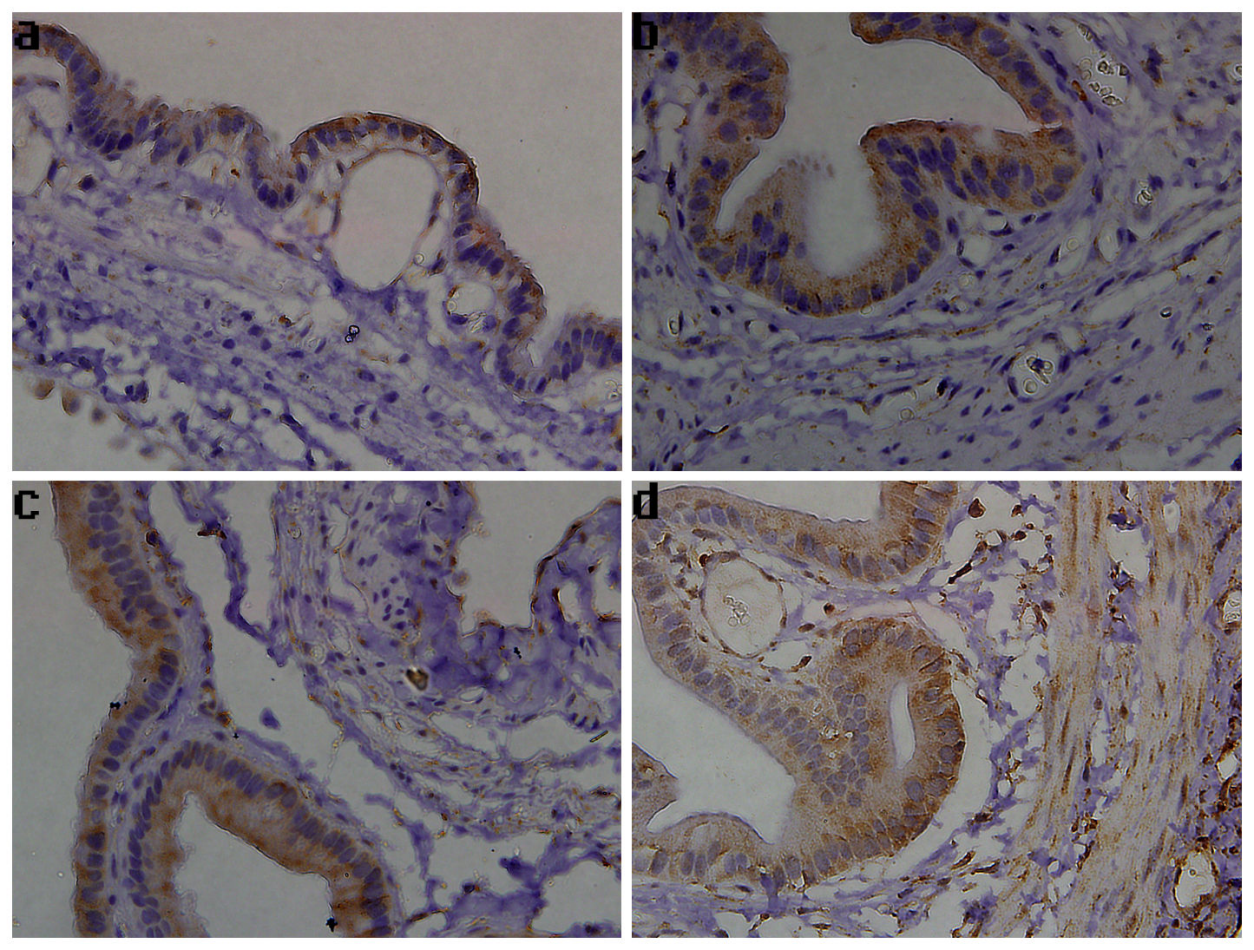
The expression of CBS in guinea pig gallbladder. Representative images of gallbladder sections stained for CBS are shown (×400). (a) Sham group, positive staining detected in the mucosal epithelial cells. (b) CBDL-1 group, positive staining detected in the mucosal epithelial cells. (c) CBDL-2 group, positive staining observed in the mucosal epithelial cells. (d) CBDL-3 group, positive staining detected in gallbladder epithelia, muscular layer, and blood vessels.

**Figure 5:**
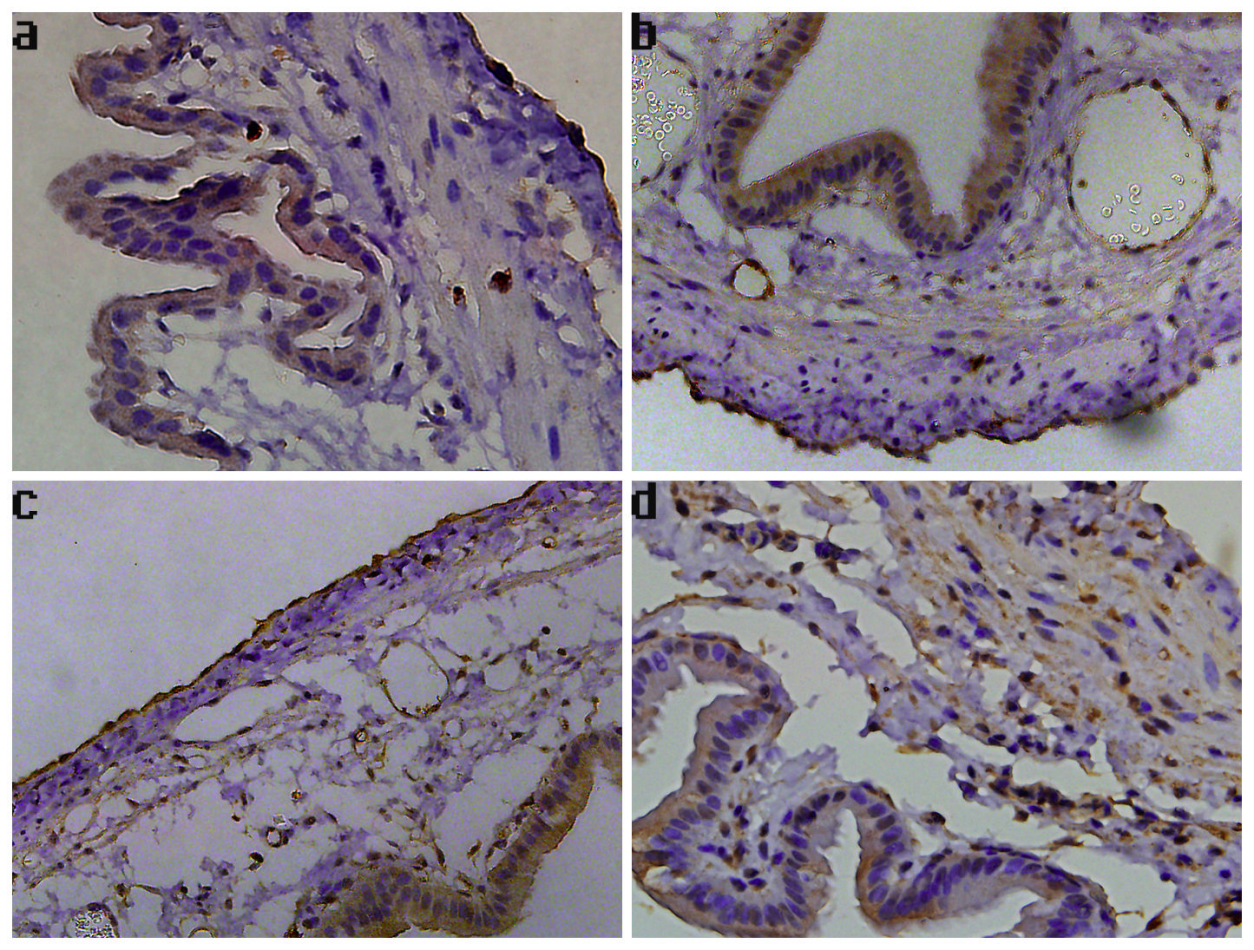
The expression of CSE in guinea pig gallbladder. Representative images of gallbladder sections stained for CSE are shown (×400). (a) Sham group, positive staining detected in the mucosal epithelial cells and serosal layer. (b) CBDL-1 group, positive staining detected in gallbladder epithelia, blood vessels, and serosal layer. (c) CBDL-2 group, positive staining detected in gallbladder epithelia, blood vessels, and serosal layer. (d) CBDL-3 group, positive staining found in gallbladder epithelia, muscular layer, and blood vessels.

Immunohistochemistry was performed to detect the expression of CBS and CSE in the gallbladder. As shown in Figure 4a-c, CBS staining was predominantly localized to mucosal epithelial cells of the gallbladder. However, the CBS-immunoreactivity was increased remarkably in tissues from days 1 to 2 post-ligation, compared to that in the sham surgical controls. In the CBDL-3 group, intense CBS staining was principally distributed in the gallbladder epithelial cells, muscular layer, and blood vessels (Figure 4d). Immunohistochemistry analysis also showed that the CSE expression was localized to mucosal epithelial cells of the gallbladder, and CBS-specific staining was also found in blood vessels and in the serosal layer (Figure 5a-c). Increased CSE immunoreactivity in tissues 3 days after CBDL was observed, particularly in gallbladder epithelia and smooth muscle cells (Figure 5d). Additionally, in the CBS-positive and CSE-positive cells, immunoreactivity for CBS and CSE was observed in the cytoplasm and plasma membrane, but not in the nucleus.

### The expression of CBS and CSE in gallbladder before and during AC

**Figure 6:**
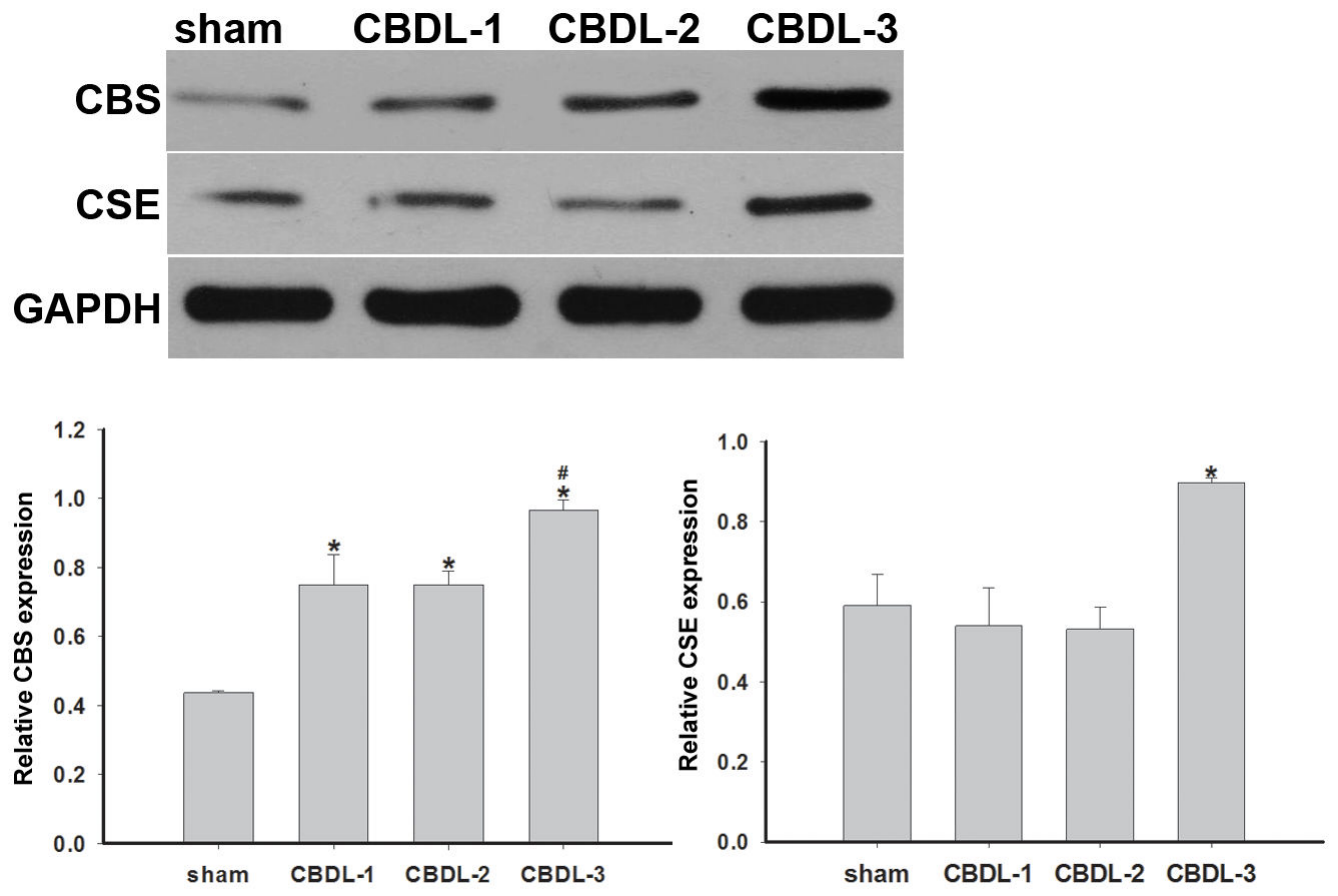
CBS and CSE protein expression in guinea pig gallbladder. CBS and CSE expression was assessed by western blot analysis, with GAPDH as the internal loading control. The bands detected by western blotting were subjected to semi-quantitative densitometric analysis and expressed as the relative CBS and CSE expression levels, after normalization to GAPDH levels. Data shown are the mean ± SD (n=12/group). *P <0.05 vs. sham; #P <0.05 vs. CBDL-1 and CBDL-2.

Western blot analysis was performed to determine protein expression of CBS and CSE. A band of about 63 kDa corresponding to CBS and a band of approximately 45 kDa corresponding to CSE were detectable in gallbladder samples. The level of CBS protein expression was significantly increased in the CBDL groups (CBDL-1, CBDL-2, and CBDL-3) compared with the sham surgical group (*P* <0.05). Moreover, the expression of CBS was remarkably elevated 3 days post-surgery compared to that in samples obtained 1 and 2 days after CBDL (*P* <0.05). There was also a significant increase in CSE expression in the CBDL-3 group compared to the sham, CBDL-1, and CBDL-2 groups (*P* <0.05). However, no statistically significant difference in CSE protein expression was found between sham, CBDL-1, and CBDL-2 groups (Figure 6).

## Discussion

The results of the present study demonstrate that CBS and CSE, the enzymes that catalyze the conversion of cysteine to H_2_S, are expressed in the gallbladder in guinea pigs. The expression of enzymes, as well as H_2_S synthesis, was significantly up-regulated during AC. Both CBS and CSE are expressed in the GI tract, including in the liver, stomach, small intestine, and colon [7]. In the present study, the expression of both CBS and CSE was detected in gallbladder tissues. CBS and CSE were predominantly localized to mucosal epithelial cells in non-inflammatory conditions. However, the expression of CBS and CSE could be detected in gallbladder epithelium, muscular layer, and blood vessels, and increased progressively with the increased inflammatory response following CBDL. In addition, that CBS was earlier raised than CSE in the course of CBDL suggested that CBS could play a major role in H_2_S generation in early inflammation of experimental AC

Previously, it has been shown that both CBS and CSE are highly expressed in the mucosa; however, CSE, but not CBS, appears to be more prominently expressed in the external muscle layers, including the myenteric plexus in the colon of the mouse [21]. Schicho R et al. [22] observed that CBS and CSE were expressed in a vast majority of guinea pig and human submucosal and myenteric neurons, but not in human colonic epithelial cells. However, in the rat colon, CBS staining was localized primarily to muscularis, mucosa, sub-mucosa and lamina propria, whereas epithelial cells were not stained with a CBS-specific antibody [23]. The present study clearly shows that both CBS and CSE are expressed in gallbladder tissues, providing evidence for the hypothesis that gallbladder mucosal epithelial cells and smooth muscle cells are able to synthesize H_2_S. These results are not consistent with those of previous reports [22,23], which may reflect the differences in tissue distribution and the model species evaluated in the studies.

There is a progressive decrease in gallbladder contractility after CBDL [2,3,24,25]. Several reports [9,11,26,27] suggest that H_2_S plays an important role in regulating gastrointestinal smooth muscle motility in mammals. Exogenous H_2_S reduces the spontaneous contractility of the guinea pig stomach and inhibits contractile activity of the ileal longitudinal muscle in rats [11,27], while endogenous H_2_S appears to be an excitatory gaseous mediator of the spontaneous contraction of gastric and ileal smooth muscle in the guinea pig [9,26]. The present study demonstrated that the level of CBS protein expression, as well as H_2_S synthesis, was significantly increased after CBDL, compared with the sham surgery, and that CBS localized to the gallbladder smooth muscle. It is possible that endogenous H_2_S is one of the factors involved in the regulation of gallbladder contractility. Zhao et al. [11] found that NaHS-induced inhibition of gastric motility in the guinea-pig is mediated via activation of ATP-sensitive potassium channels. Han et al. [9] demonstrated that the excitatory function of endogenous H_2_S on gastric motility in mice is mediated via inhibition of delayed rectifier K^+^ current. Further studies are needed to explore the mechanisms responsible for the effects of H_2_S on gallbladder contractility during AC.

In this study, we also found prominent inflammatory cell infiltration in the lamina propria, and a marked vascular congestion, with splaying of the muscle fibers, after CBDL, consistent with the results of previous studies [2,3,24,25]. It has been demonstrated that H_2_S is generated at sites of inflammation and can suppress leukocyte adherence to the vascular endothelium and reduce leukocyte infiltration and edema formation [12,28,29]. A progressive inflammatory response, in this study, was accompanied with an increase in CBS and CSE expression in blood vessel endothelium, which may suggest an anti-inflammatory role for endogenous H_2_S in the context of inflammation during AC. However, there are conflicting data regarding the roles of H_2_S in the regulation of inflammation, including hindpaw edema [15], AP [16], LPS-induced endotoxemia [17] and CLP-induced sepsis [18,19]. H_2_S activates the transcriptional activity of nuclear factor (NF)-κB in AP to promote inflammation [19,30]. H_2_S inhibits NF-κB in atherosclerotic progression to suppress inflammation [31]. Inflammation models and the organ of origin of H_2_S may be accountable for the discrepancies in the role of H_2_S in inflammation reported in these studies. 

In conclusion, gallbladder cells express CBS and CSE, and the expression of these enzymes, as well as H_2_S synthesis, was up-regulated in the context of inflammation during AC. This study provides the basis for further investigations of the role of endogenously-produced H_2_S in the gallbladder and the effects of H_2_S on inflammatory responses and gallbladder contractility during AC.

## References

[1] Gomez-Pinilla PJ, Camello PJ, Tresguerres JA, Pozo MJ (2010) Tempol protects the gallbladder against ischemia/reperfusion. J Physiol Biochem 66: 161-172. doi:10.1007/s13105-010-0021-y. PubMed: 20571964. 20571964

[2] Soylu S, Aydin C, Bagcivan I, Yildirim S, Koyuncu A et al. (2009) Effects of NO/L-arginine pathway on gallbladder contractility in bile duct ligated guinea pigs. J Surg Res 155: 70-76. doi:10.1016/j.jss.2008.08.002. PubMed: 19394644. 19394644

[3] Parkman HP, Bogar LJ, Bartula LL, Pagano AP, Thomas RM et al. (1999) Effect of experimental acalculous cholecystitis on gallbladder smooth muscle contractility. Dig Dis Sci 44: 2235-2243. doi:10.1023/A:1026600603121. PubMed: 10573368. 10573368

[4] Łowicka E, Bełtowski J (2007) Hydrogen sulfide (H_2_S)-the third gas of interest for pharmacologists. Pharmacol Rep 59: 4-24. PubMed: 17377202. 17377202

[5] Renga B (2011) Hydrogen sulfide generation in mammals: the molecular biology of cystathionine-β-synthase (CBS) and cystathionine-γ-lyase (CSE). Inflamm Allergy Drug Targets 10: 85-91. doi:10.2174/187152811794776286. PubMed: 21275900. 21275900

[6] Li H, Mani S, Cao W, Yang G, Lai C et al. (2012) Interaction of hydrogen sulfide and estrogen on the proliferation of vascular smooth muscle cells. PLOS ONE 7: e41614. doi:10.1371/journal.pone.0041614. PubMed: 22870237. 22870237 PMC3411693

[7] Wang R (2010) Hydrogen sulfide: the third gasotransmitter in biology and medicine. Antioxid Redox Signal 12: 1061-1064. doi:10.1089/ars.2009.2938. PubMed: 19845469. 19845469

[8] Wang R (2012) Physiological implications of hydrogen sulfide: a whiff exploration that blossomed. Physiol Rev 92: 791-896. doi:10.1152/physrev.00017.2011. PubMed: 22535897. 22535897

[9] Han YF, Huang X, Guo X, Wu YS, Liu DH et al. (2011) Evidence that endogenous hydrogen sulfide exerts an excitatory effect on gastric motility in mice. Eur J Pharmacol 673: 85-95. doi:10.1016/j.ejphar.2011.10.018. PubMed: 22047765. 22047765

[10] Linden DR (2013) Hydrogen Sulfide Signaling in the Gastrointestinal Tract. Antioxid Redox Signal. [in print]. 10.1089/ars.2013.5312 PMC3910452 23582008

[11] Zhao P, Huang X, Wang ZY, Qiu ZX, Han YF et al. (2009) Dual effect of exogenous hydrogen sulfide on the spontaneous contraction of gastric smooth muscle in guinea-pig. Eur J Pharmacol 616: 223-228. doi:10.1016/j.ejphar.2009.05.014. PubMed: 19470382. 19470382

[12] Li L, Bhatia M, Moore PK (2006) Hydrogen sulphide--a novel mediator of inflammation? Curr Opin Pharmacol 6: 125-129. doi:10.1016/j.coph.2005.10.007. PubMed: 16487749. 16487749

[13] Cui J, Liu L, Zou J, Qiao W, Liu H et al. (2013) Protective effect of endogenous hydrogen sulfide against oxidative stress in gastric ischemia-reperfusion injury. Exp Ther Med 5: 689-694. PubMed: 23403765. 23403765 10.3892/etm.2012.870 PMC3570130

[14] Yonezawa D, Sekiguchi F, Miyamoto M, Taniguchi E, Honjo M et al. (2007) A protective role of hydrogen sulfide against oxidative stress in rat gastric mucosal epithelium. Toxicology 241: 11-18. doi:10.1016/j.tox.2007.07.020. PubMed: 17825973. 17825973

[15] Bhatia M, Sidhapuriwala J, Moochhala SM, Moore PK (2005) Hydrogen sulphide is a mediator of carrageenan-induced hindpaw oedema in the rat. Br J Pharmacol 145: 141-144. doi:10.1038/sj.bjp.0706186. PubMed: 15753944. 15753944 PMC1576135

[16] Bhatia M, Wong FL, Fu D, Lau HY, Moochhala SM et al. (2005) Role of hydrogen sulfide in acute pancreatitis and associated lung injury. FASEB J 19: 623-625. PubMed: 15671155. 15671155 10.1096/fj.04-3023fje

[17] Li L, Bhatia M, Zhu YZ, Zhu YC, Ramnath RD et al. (2005) Hydrogen sulfide is a novel mediator of lipopolysaccharide-induced inflammation in the mouse. FASEB J 19: 1196-1198. PubMed: 15863703. 15863703 10.1096/fj.04-3583fje

[18] Zhang H, Zhi L, Moochhala S, Moore PK, Bhatia M (2007) Hydrogen sulfide acts as an inflammatory mediator in cecal ligation and puncture-induced sepsis in mice by upregulating the production of cytokines and chemokines via NF-kappaB. Am J Physiol Lung Cell Mol Physiol 292: L960-L971. PubMed: 17209138. 17209138 10.1152/ajplung.00388.2006

[19] Zhang H, Hegde A, Ng SW, Adhikari S, Moochhala SM et al. (2007) Hydrogen sulfide up-regulates substance P in polymicrobial sepsis-associated lung injury. J Immunol 179: 4153-4160. PubMed: 17785854. 17785854 10.4049/jimmunol.179.6.4153

[20] Hui Y, Du J, Tang C, Bin G, Jiang H (2003) Changes in arterial hydrogen sulfide (H(2)S) content during septic shock and endotoxin shock in rats. J Infect 47: 155-160. doi:10.1016/S0163-4453(03)00043-4. PubMed: 12860150. 12860150

[21] Linden DR, Sha L, Mazzone A, Stoltz GJ, Bernard CE et al. (2008) Production of the gaseous signal molecule hydrogen sulfide in mouse tissues. J Neurochem 106: 1577-1585. PubMed: 18513201. 18513201 10.1111/j.1471-4159.2008.05502.x PMC2836856

[22] Schicho R, Krueger D, Zeller F, Von Weyhern CW, Frieling T et al. (2006) Hydrogen sulfide is a novel prosecretory neuromodulator in the Guinea-pig and human colon. Gastroenterology 131: 1542-1552. doi:10.1053/j.gastro.2006.08.035. PubMed: 17101327. 17101327

[23] Martin GR, McKnight GW, Dicay MS, Coffin CS, Ferraz JG et al. (2010) Hydrogen sulphide synthesis in the rat and mouse gastrointestinal tract. Dig Liver Dis 42: 103-109. doi:10.1016/S1590-8658(10)60117-X. PubMed: 19570733. 19570733

[24] Parkman HP, James AN, Bogar LJ, Bartula LL, Thomas RM et al. (2000) Effect of acalculous cholecystitis on gallbladder neuromuscular transmission and contractility. J Surg Res 88: 186-192. doi:10.1006/jsre.1999.5788. PubMed: 10644487. 10644487

[25] Parkman HP, James AN, Thomas RM, Bartula LL, Ryan JP et al. (2001) Effect of indomethacin on gallbladder inflammation and contractility during acute cholecystitis. J Surg Res 96: 135-142. doi:10.1006/jsre.2001.6082. PubMed: 11181007. 11181007

[26] Teague B, Asiedu S, Moore PK (2002) The smooth muscle relaxant effect of hydrogen sulphide in vitro: evidence for a physiological role to control intestinal contractility. Br J Pharmacol 137: 139-145. doi:10.1038/sj.bjp.0704858. PubMed: 12208769. 12208769 PMC1573483

[27] Nagao M, Linden DR, Duenes JA, Sarr MG (2011) Mechanisms of action of the gasotransmitter hydrogen sulfide in modulating contractile activity of longitudinal muscle of rat ileum. J Gastrointest Surg 15: 12-22. doi:10.1007/s11605-010-1306-8. PubMed: 21082276. 21082276 PMC3046388

[28] Whiteman M, Armstrong JS, Chu SH, Jia-Ling S, Wong BS et al. (2004) The novel neuromodulator hydrogen sulfide: an endogenous peroxynitrite 'scavenger'? J Neurochem 90: 765-768. doi:10.1111/j.1471-4159.2004.02617.x. PubMed: 15255956. 15255956

[29] Zanardo RC, Brancaleone V, Distrutti E, Fiorucci S, Cirino G et al. (2006) Hydrogen sulfide is an endogenous modulator of leukocyte-mediated inflammation. FASEB J 20: 2118-2120. doi:10.1096/fj.06-6270fje. PubMed: 16912151. 16912151

[30] Tamizhselvi R, Shrivastava P, Koh YH, Zhang H, Bhatia M (2011) Preprotachykinin-A gene deletion regulates hydrogen sulfide-induced toll-like receptor 4 signaling pathway in cerulein-treated pancreatic acinar cells. Pancreas 40: 444-452. doi:10.1097/MPA.0b013e31820720e6. PubMed: 21289528. 21289528

[31] Wang Y, Zhao X, Jin H, Wei H, Li W et al. (2009) Role of hydrogen sulfide in the development of atherosclerotic lesions in apolipoprotein E knockout mice. Arterioscler Thromb Vasc Biol 29: 173-179. doi:10.1161/ATVBAHA.108.179333. PubMed: 18988885. 18988885

